# Lightweight Infrared Image Denoising Method Based on Adversarial Transfer Learning

**DOI:** 10.3390/s24206677

**Published:** 2024-10-17

**Authors:** Wen Guo, Yugang Fan, Guanghui Zhang

**Affiliations:** 1 Faculty of Information Engineering and Automation, Kunming University of Science and Technology, Kunming 650500, China; wenguo@stu.kust.edu.cn (W.G.); ghzhang@kust.edu.cn (G.Z.); 2 Yunnan Key Laboratory of Intelligent Control and Application, Kunming University of Science and Technology, Kunming 650500, China

**Keywords:** infrared image denoising, structural reparameterization, transfer learning, adversarial learning, deep learning

## Abstract

A lightweight infrared image denoising method based on adversarial transfer learning is proposed. The method adopts a generative adversarial network (GAN) framework and optimizes the model through a phased transfer learning strategy. In the initial stage, the generator is pre-trained using a large-scale grayscale visible light image dataset. Subsequently, the generator is fine-tuned on an infrared image dataset using feature transfer techniques. This phased transfer strategy helps address the problem of insufficient sample quantity and variety in infrared images. Through the adversarial process of the GAN, the generator is continuously optimized to enhance its feature extraction capabilities in environments with limited data. Moreover, the generator structure incorporates structural reparameterization technology, edge convolution modules, and progressive multi-scale attention block (PMAB), significantly improving the model’s ability to recognize edge and texture features. During the inference stage, structural reparameterization further optimizes the network architecture, significantly reducing model parameters and complexity and thereby improving denoising efficiency. The experimental results of public and real-world datasets demonstrate that this method effectively removes additive white Gaussian noise from infrared images, showing outstanding denoising performance.

## 1. Introduction

Infrared image denoising is an essential part of infrared signal processing. As a key preprocessing step for advanced visual tasks such as multimodal image fusion and night target detection, infrared image denoising has garnered extensive attention [1]. Infrared images are captured by the detector of an infrared thermal imaging camera, which detects the infrared radiation energy emitted by objects, converts it into electrical signals, amplifies and processes these signals, and then converts them into standard video signals displayed as thermal maps on a monitor. However, infrared imaging is easily affected by heat conduction effects and air scattering, resulting in a low signal-to-noise ratio (SNR), poor contrast, and severe noise interference [2]. These issues are significant obstacles that limit the widespread application of infrared imaging technology. Therefore, infrared image denoising is crucial for the practical application of infrared technology.

Traditional image denoising methods are mainly divided into two categories: spatial domain and transform domain methods. In the field of infrared image denoising, spatial domain methods, such as pixel value smoothing [3] and filtering [4], are widely applied due to their computational simplicity and high efficiency. These methods reduce noise by calculating the mean or median of neighboring pixels. However, this process often leads to the excessive smoothing of image details and edge information, which reduces the local contrast and clarity of the image. In infrared images, fine temperature gradients and subtle edge features are critical, making this limitation of spatial domain methods even more evident in infrared image processing. In contrast, transform domain methods, such as block-matching and 3D filtering (BM3D) [5] and wavelet transform (WT) [6], decompose the image into components at different scales and orientations and remove noise based on the different characteristics of the noise and the image signal in the frequency or wavelet domain. These methods excel in preserving image structure and texture, especially in retaining detailed and complex texture information. However, transform domain methods do not always accurately distinguish between signal and noise and may introduce artifacts or cause the loss of specific image features during reconstruction, which can ultimately affect the overall quality of the denoised image.

In recent years, deep learning technology has achieved significant progress in image denoising due to its excellent feature learning capabilities [7]. Zhang et al. [8] proposed the deep residual convolutional neural network denoising algorithm (DnCNN), which uses residual learning to predict the difference between noise and the underlying clean image for denoising. Based on this, Zhang et al. [9] proposed FFDNet, which introduces a noise level map as an additional input parameter, enabling the network to more precisely denoise images at different noise levels. Li et al. [10] proposed a denoising method that enhances the model’s feature extraction capability by combining second-order attention mechanisms and non-local modules. Hu et al. [11] proposed a denoising method based on a symmetric multi-scale (SM) encoder-decoder structure utilizing residual learning. Lyu et al. [12] proposed the STPFD, which effectively addresses the challenge of detail preservation in high-noise environments by integrating the non-subsampled Shearlet transform (NSST) with a dual convolutional neural network. Yang et al. [13] introduced multi-level residual blocks into their GAN to improve feature learning capabilities. However, this improvement increased computational complexity. As a result, it limited the denoising efficiency.

Despite significant progress in denoising performance with deep learning models, they still require a large amount of training data to optimize the model. However, in practical applications, the quantity and diversity of clean infrared image samples are often insufficient, leading to most existing methods relying heavily on large amounts of visible light images for training. Although both infrared and visible light images can be represented in grayscale, there are fundamental differences in their physical properties and visual representations [14]. This training strategy significantly limits the performance of models in handling pure infrared image denoising tasks. Additionally, current deep learning methods face a trade-off between denoising performance and resource consumption—improving denoising performance often comes with high computational resource demands, which slows down processing speed and affects the efficiency and deployment of the overall system. Therefore, it is necessary to explore denoising methods that remain effective under data-limited conditions to enhance the applicability and efficiency of the model. To address the aforementioned issues, this paper proposes the following innovations:

(1) A lightweight GAN architecture is proposed, with the core of utilizing structural reparameterization technology to equivalently transform the multi-branch structure of the generator into a single-branch structure during the inference stage, effectively reducing the model’s computational resource consumption and achieving an efficient lightweight design.
(2) Structural reparameterization edge convolution block (SRECB) and progressive multi-scale attention block (PMAB) are introduced into the generator, allowing the generator to accurately recognize edge and texture information in the source image, significantly improving the model’s denoising performance.
(3) A decision-making mechanism for deep feature space parameter transfer in infrared images is constructed, effectively enhancing the representation capability of infrared image features in small sample environments.

## 2. Method

**Figure 1:**
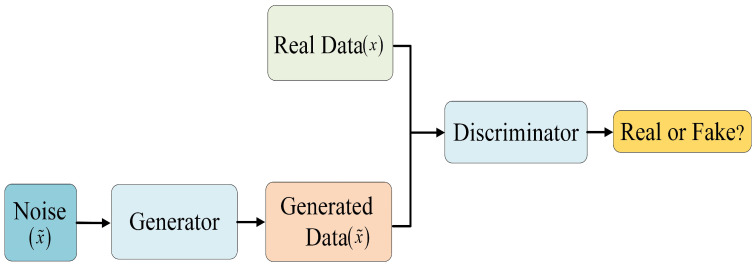
Overall network architecture.

GAN [15] has been widely applied to various image restoration tasks such as image denoising [16] and image dehazing [17], showing good performance and restoration effects. Based on this framework, this paper designs a novel lightweight denoising model. Figure 1 shows the overall architecture of the network, which includes a generator and a discriminator. The generator aims to produce clear images from noisy input images, while the discriminator evaluates the differences between these generated images and actual noise-free images. The adversarial process is represented as follows:

(1) $$\min\max_{D}V_{GAN}(G,D)=E_{x∼P_{\mathrm{data}}}[\log D(x)]+E_{\tilde{x}∼P_{G}}[\log(1-D(G(\tilde{x})))]$$

During training, the generator attempts to generate images that can deceive the discriminator, while the discriminator tries to distinguish between the generated images and real images. When the loss functions of the generator and discriminator stabilize, it indicates that the model has converged, and the generator can produce high-quality images, while the discriminator finds it challenging to distinguish between these images and real images. The design of the network structure for the generator and the discriminator, as well as the decision-making for feature transfer, are detailed below.

### 2.1. Generator Network

**Figure 2:**
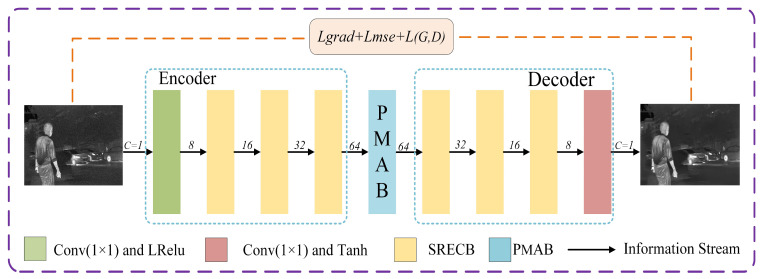
Schematic diagram of the generator.

As shown in Figure 2, the generator mainly consists of three parts: an encoder, a progressive multi-scale attention block (PMAB), and a decoder. The encoder and decoder have similar structures, composed of structured reparameterized edge convolution blocks (SRECB). During training, the noisy input image is first subjected to shallow feature extraction through a 1 × 1 convolution, producing preliminary features. These preliminary features are then mapped to a high-dimensional feature space by the edge convolution module. Next, the progressive multi-scale attention block (PMAB) further explores and integrates high-dimensional feature relationships, optimizing feature expression capabilities and providing richer and more refined feature information for the decoder. Ultimately, the decoder uses these finely processed features to reconstruct a clear output image.

### 2.2. Structurally Reparameterizable Edge Convolution Block (SRECB)

**Figure 3:**
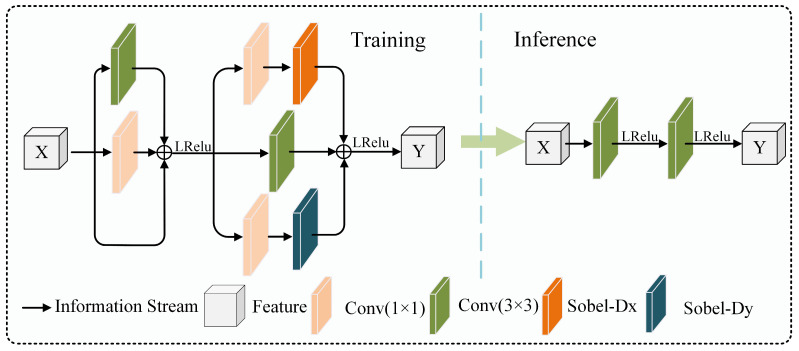
Structurally reparameterizable edge convolution block.

Many existing infrared denoising methods rely on complex network architectures to enhance performance; however, this often significantly increases computational complexity. In ACNet [18], Ding et al. first proposed structural equivalence transformation, which converts multi-branch structures into single-branch structures, significantly improving convolutional network performance. Following this, structural reparameterization technology has demonstrated significant effectiveness in various vision tasks, such as super-resolution reconstruction [19] and object detection [20], providing new insights into the field of infrared denoising. Building on this, this paper designs a structurally reparameterizable edge convolution block, whose network structure is illustrated in Figure 3.

The structurally reparameterizable edge convolution block (SRECB) consists of two main parts. The first part contains a 3 × 3 convolution layer, a 1 × 1 convolution layer, and an identity mapping layer. During training, the 3 × 3 convolution layer uses its larger convolution kernel to capture spatial information, such as textures and edges. The 1 × 1 convolution layer operates on each pixel to optimize the depth of the feature map, refining and enhancing the captured features and improving the accuracy and consistency of feature extraction. The added identity mapping layer enhances the network’s stability and completeness in handling deep features. During the inference phase, based on the linear additivity of convolution, the multi-branch convolution can be equivalently represented as a single convolution, as shown by the following equations:

(2) $$Conv\left(X,\left(W_{1}+W_{2}+W_{3}\right)\right)=Conv\left(X,\left(W_{1}\right)\right)+Conv\left(X,\left(W_{2}\right)\right)+Conv\left(X,\left(W_{3}\right)\right)$$

In the equation, Conv represents the convolution operation, X is the input feature, and W is the convolution parameter, including the weights and biases of the convolution. Therefore, using this property, the equivalent operation process for the first part is shown in Equations (3)–(5).

(3) $$F_{n}=(K_{3}*X+B_{3})+(K_{1}*X+B_{1})+(K_{0}*X)$$

(4) $$F_{n}=(K_{3}+dil\left(K_{1}\right)+dil\left(K_{0}\right))*X+(B_{3}+B_{1})$$

(5) $$F_{n}=K_{e}*X+B_{e}$$

where X represents the input features, $F_{n}$ represents the output features, $K_{1}$ and $B_{1}$ represent the weights and biases of the 1 × 1 convolution, $K_{3}$ and $B_{3}$ represent the weights and biases of the 3 × 3 convolution, and $K_{0}$ represents the identity mapping of the first part (equivalent to an identity matrix convolution). $K_{e}$ and $B_{e}$ are the weights and biases of the equivalent 3 × 3 convolution. Additionally, dil represents the dilation operation.

Unlike the first part, the second part of the network embeds a predefined Sobel filter to effectively extract edge information from images. This filter captures the first-order spatial derivatives, i.e., local variations in image brightness, thereby highlighting edges. During the training phase, the features extracted by the first part of the network are processed in the edge operator branch through a 1 × 1 convolution layer to effectively aggregate information from different channels, enabling preliminary feature extraction and processing. These features are then subjected to edge detection using horizontal and vertical Sobel filters. The introduced learnable scaling factor allows the network to adjust the filter’s response strength during training, thus adapting to different image characteristics and denoising requirements. The 3 × 3 convolution branch is used to maintain the fundamental performance of the input features from the previous part. The equivalent operation process for the second part’s branches is shown in Equations (6)–(9).

(6) $$D_{X}=\left[\begin{array}{ccc} +1 & 0 & -1\\ +2 & 0 & -2\\ +1 & 0 & -1 \end{array}\right]\;and\;D_{y}=\left[\begin{array}{ccc} +1 & +2 & +1\\ 0 & 0 & 0\\ -1 & -2 & -1 \end{array}\right]$$

(7) $$F_{Dy}=(s_{Dy}\cdot D_{y})⊗(K_{y}*F_{n}+B_{y})+B_{Dy}$$

(8) $$F_{Dx}=(s_{Dx}\cdot D_{x})⊗(K_{x}*F_{n}+B_{x})+B_{Dx}$$

(9) $$F_{3\times 3}=(K_{3}*F_{n}+B_{3})$$

$K_{x}$ and $B_{x}$ represent the weights and biases of the 1 × 1 convolution in the horizontal branch, while $K_{y}$ and $B_{y}$ represent those in the vertical branch. $K_{3}$ and $B_{3}$ represent the weights and biases of the 3 × 3 convolution. $D_{x}$, $D_{y}$, $B_{Dx}$, and $B_{Dy}$ represent the weights and biases of the Sobel operators in the horizontal and vertical directions, respectively. Similarly, $s_{Dx}$ and $s_{Dy}$ are the corresponding learnable scaling factors. The symbols ⊗ and ∗ denote depth-wise convolution (DWConv) and standard convolution, respectively, and ⋅ denotes channel-wise multiplication. The edge information features extracted by the edge operator branch and the total feature information extracted by the 3 × 3 convolution branch are as follows:

(10) $${F^{\prime }}_{n}=F_{Dx}+F_{Dy}+F_{3\times 3}$$

${F^{\prime }}_{n}$ is the overall output of this module. After training is completed, similar to the first part, the structure of the second part is equivalently transformed into a 3 × 3 convolution layer during the inference phase. Thus, in the inference stage, only this reparameterized 3 × 3 convolution is needed to achieve the same effect. The equivalent operation process is shown in Equations (11)–(13).

(11) $$K_{s}=K_{x}⊗K_{Dx}+K_{y}⊗K_{Dy}+K_{3}$$

(12) $$B_{s}=\left(K_{Dx}⊗rep\left(B_{x}\right)+B_{Dx}\right)+\left(K_{Dy}⊗rep\left(B_{y}\right)+B_{Dy}\right)+B_{3}$$

(13) $${F^{\prime }}_{n}=K_{s}*F_{n}+B_{s}$$

In the equation, $K_{s}$ and $B_{s}$ are the weights and biases after the equivalent transformation, and rep denotes the broadcasting operation, which is intended to replicate the 1 × 1 bias to match the size of the 3 × 3 bias.

### 2.3. Progressive Multi-Scale Attention Block (PMAB)

**Figure 4:**
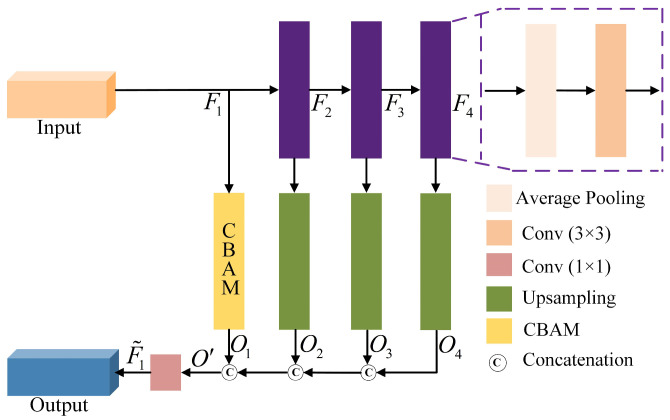
The structure of progressive multi-scale attention block (PMAB).

To further enhance feature expression capabilities, a progressive multi-scale attention block (PMAB) is proposed. PMAB integrates an attention branch based on the convolutional block attention module (CBAM) and a multi-scale feature extraction branch to optimize the model’s ability to capture and utilize critical visual information. The structure of the PMAB is shown in Figure 4.

**Figure 5:**
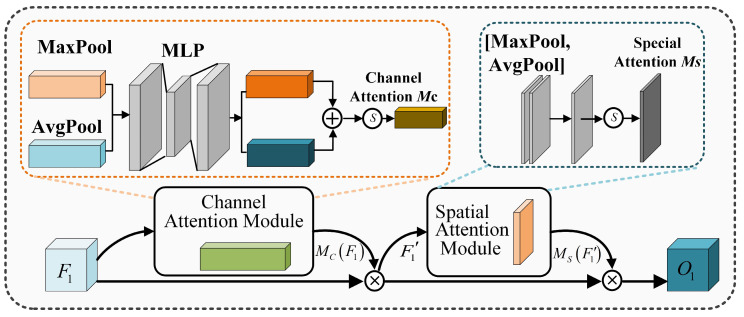
The structure of convolutional block attention module (CBAM).

The CBAM branch adjusts the importance of features across both channel and spatial dimensions, further enhancing the model’s ability to focus on key features. The structure of the CBAM module is shown in Figure 5. The CBAM module first identifies and enhances the feature channels that contribute to task performance through a channel attention mechanism. Then, the spatial attention mechanism highlights useful spatial regions, allowing the overall network to focus more on features that are highly discriminative for the final task. The process is described by the following equations:

(14) $${F^{\prime }}_{1}=M_{C}\left(F_{1}\right)\times F_{1}$$

(15) $$O_{1}=M_{s}\left({F^{\prime }}_{1}\right)\times {F^{\prime }}_{1}$$

where $M_{C}\left(F_{1}\right)$ and $M_{s}\left({F^{\prime }}_{1}\right)$ represent the channel and spatial attention weights, respectively, $F_{1}$ represents the input features, and $O_{1}$ represents the output features.

The multi-scale feature extraction branch performs down-sampling and up-sampling on the input feature $F_{1}$, obtaining three different levels of features $O_{2}$, $O_{3}$, and $O_{4}$. This process effectively captures contextual information at different scales, enabling the model to gain a more comprehensive understanding of the image content, thereby enhancing the diversity and robustness of the feature representation. The outputs from the two branches are then merged using a channel concatenation strategy to ensure that the information obtained from each branch is fully utilized, followed by a 1 × 1 convolution for channel compression to generate enhanced feature representations. This process is illustrated in Equation (16).

(16) $${\tilde{F}}_{1}=Conv\left(Concat\left\{O_{1},O_{2},O_{3},O_{4}\right\}\right)$$

In the equation, ${\tilde{F}}_{1}$ represents the enhanced features output by the progressive multi-scale attention block (PMAB). These features are then reconstructed by the decoder into a clean, denoised image.

### 2.4. Discriminator Design

**Figure 6:**
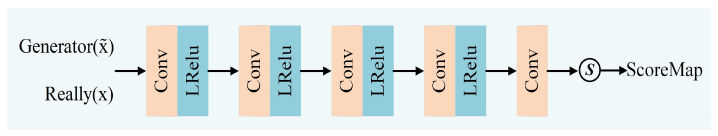
The structure of discriminator.

As shown in Figure 6, the discriminator network adopts the structure of PatchGAN [21], which aims to enhance the model’s ability to distinguish details and local features by judging each local patch of the image. The discriminator network comprises five layers of convolutional neural networks. The input image is a generated or real image with a size of 1 × 128 × 128. From the first layer to the fourth layer, the convolution kernel size is 3 × 3, and the stride is set to 2. The number of channels in each layer is 16, 32, 64, and 128, respectively. The final layer does not perform down-sampling and outputs a score map with 256 channels and a size of 8 × 8. The output score map represents the probability that the input image is a real image or a generated image.

### 2.5. Transfer Strategy Based on Model Parameters

**Figure 7:**
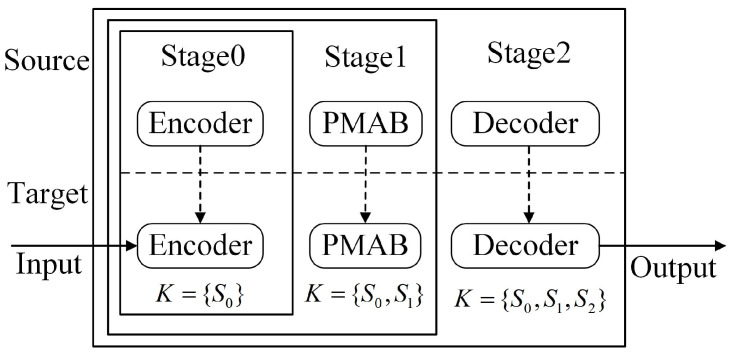
Schematic diagram of model parameter transfer.

Transfer learning is an effective technique for sharing knowledge between different tasks, which significantly reduces the size of the dataset required to construct a sufficiently separable feature space in the target task [22]. This paper proposes a phased transfer learning strategy aimed at improving feature expression capabilities in small sample tasks. In the initial stage, the generator is pre-trained on the source domain dataset until the model converges. Then, the pre-trained generator network is trained in the target domain in three parts: the encoder, the progressive multi-scale attention block (PMAB), and the decoder. The transfer learning process is shown in Figure 7. First, the progressive multi-scale attention block (PMAB) and decoder parameters are frozen, and only the encoder part’s parameters are trained. Next, the progressive multi-scale attention block’s parameters are unfrozen while continuing to freeze the decoder’s parameters, training both the encoder and progressive multi-scale attention module. Finally, the decoder’s parameters are unfrozen, and the entire network is trained jointly.

### 2.6. Model Optimization

The model is optimized by using a weighted sum of gradient loss, mean square error (MSE) loss, and adversarial loss as the objective function to train the generator. Gradient loss focuses on regions in the image where pixel values change, particularly edges and details. By minimizing the difference between the generated image and the real image in these regions, the image’s edges and details are enhanced. The gradient loss is described by the following equation:

(17) $$L_{grand}=\frac{1}{N}\sum_{i=1}^{N}\left\|\left|\nabla X_{i}|-|\nabla G({\tilde{X}}_{i})\right|\right\|$$

where N represents the number of samples, ∇ is the gradient operator that measures the texture information in the image, $x_{i}$ represents the real sample, and $G({\tilde{x}}_{i})$ denotes the high-quality image generated by the generator.

MSE loss primarily measures the difference between the generated image and the real image at the pixel level, thereby improving the quality of the generated image. The MSE loss is described by the following equation:

(18) $$L_{MSE}=\frac{1}{N}\overset{N}{\underset{i=1}{\sum }}{\left(G({\tilde{x}}_{i})-x_{i}\right)}^{2}$$

The adversarial loss is described by the following equation, where the least squares loss is used instead of the cross-entropy loss in the original GAN. For the discriminator, the objective is to maximize the probability of distinguishing real images as true and generated images as false, with the label for real images set to 1 and generated images to 0. For the generator, the goal is to generate images as close to the original image as possible; thus, the label for real images is set to 1.

(19) $$\begin{array}{cc} L_{A}(G,D): & \min_{D}=\frac{1}{2}E[{\left(D(x)-1\right)}^{2}]+\frac{1}{2}E[{\left(D(G(\tilde{x}))\right)}^{2}]\\ & \min_{G}=E[D{\left(G(\tilde{x})-1\right)}^{2}] \end{array}$$

Thus, the total loss function is as follows:

(20) $$L_{total}=\lambda_{1}L_{grand}+\lambda_{2}L_{MSE}+\lambda_{3}L_{A}(G,D)$$

where $\lambda_{1}$, $\lambda_{2}$, and $\lambda_{3}$ are the balancing parameters.

## 3. Experimental Analysis

### 3.1. Experimental Setup

In the initial stage, the model is pre-trained on a large grayscale visible light image dataset [23]. Once the model converges on the visible light dataset, the feature space parameters of the source domain images are extracted from this model. These parameters serve as the foundation for transfer learning, helping the model quickly adapt to a new data domain in subsequent stages. After pre-training, the model is further trained on 100 infrared images [24], with 80 images used for training and 20 images used for testing. The 80 training images are augmented through flipping and cropping to generate 20,000 sub-images with a resolution of 128 × 128 pixels. To simulate different noise environments that might be encountered in practical applications, these sub-images are divided into four groups, each injected with additive white Gaussian noise (AWGN) of different levels: σ = 15, 25, 50, and a random variable ranging from 0 to 25, to obtain corresponding low-quality images. The entire training process is set to 300 epochs, uniformly divided into three stages (Stage0, Stage1, Stage2), with each stage corresponding to different transfer learning strategies and network components. The hyperparameters $\lambda_{1}$, $\lambda_{2}$, and $\lambda_{3}$ are set to 3, 10, and 0.5, respectively. The batch size is set to 32, the learning rate is 0.001, and the optimizer is ADAM. The experiments in this paper were conducted on a GeForce RTX 4090 24G GPU using the Pytorch framework.

### 3.2. Experiments on Public Datasets

In this section, the proposed model is comprehensively evaluated and compared with seven advanced methods: BM3D [5], DnCNN [8], FFDNet [9], SMEDS [11], CBDNet [25], IE-CGAN [26], and FEUNet [27]. The evaluation method combines subjective and objective assessments: subjective evaluation involves visually comparing the denoised image quality; objective evaluation quantifies the restoration of image quality and structural integrity using peak signal-to-noise ratio (PSNR) and structural similarity index (SSIM).

Table 1 presents the average test results across different noise levels, with bold values indicating the best performance and underlined values representing the second best. From the data, the proposed method demonstrates excellent denoising performance across different noise levels (σ = 15, 25, 50). Specifically, at a low noise level (σ = 15), our model achieved the highest PSNR (34.389) and SSIM (0.829), indicating superior performance in preserving image quality and structural similarity compared to other models. With increased noise levels (σ = 25, 50), the proposed method continues to maintain high PSNR and SSIM values. Notably, the average PSNR and SSIM of the proposed method at all three noise levels are superior to state-of-the-art methods, highlighting the method’s stability and superior performance.

**Figure 8:**
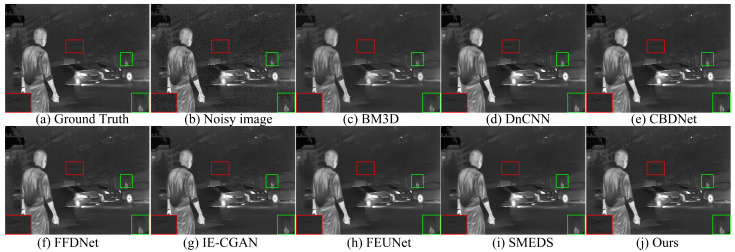
Denoising results of different methods on the AWGN with a variance of σ = 15.

**Figure 9:**
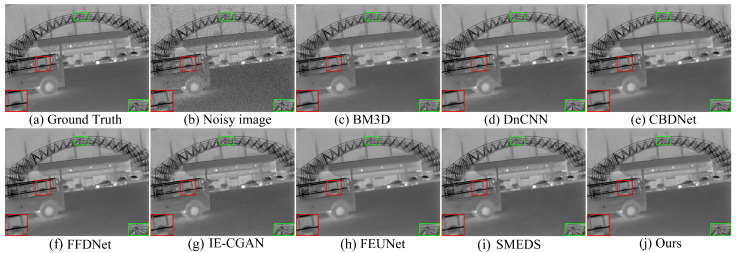
Denoising results of different methods on the AWGN with a variance of σ = 25.

**Figure 10:**
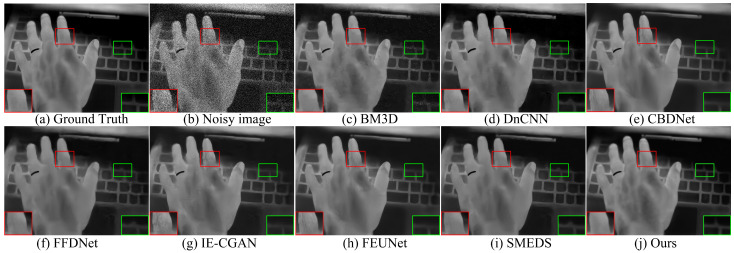
Denoising results of different methods on the AWGN with a variance of σ = 50.

Figure 8, Figure 9 and Figure 10 show the visual results at different noise levels. Specifically, Figure 8 illustrates that under a low noise level, most methods are capable of completing the denoising task, but the proposed method produces images with better contrast. In the enlarged texture details, our method better preserves the texture of tree trunks and the contours of pedestrians, while other methods retain some residual noise. Figure 9 demonstrates that as the noise level increases, the BM3D and DnCNN methods cause the background of the denoised images to become blurry. In the enlarged texture details, the proposed method better preserves the texture of arches and the contours of cars, whereas other methods result in a significant loss of texture details. In Figure 10, when the noise level increases to σ = 50, the proposed method better preserves the detailed texture of the keyboard and the clear shape of the hand, while the images restored by other methods exhibit varying degrees of blurring. These results indicate that the proposed method achieves the best visual effect even under high noise conditions.

In this study, the proposed method performed excellently in both subjective and objective evaluations. In contrast, other methods either resulted in blurring after denoising or lost a significant amount of texture details. These results fully demonstrate the advantages and potential of the proposed method in image denoising tasks, as it not only provides superior denoising performance but also preserves richer image details and structure visually.

### 3.3. Experiments on Real-World Datasets

**Figure 11:**
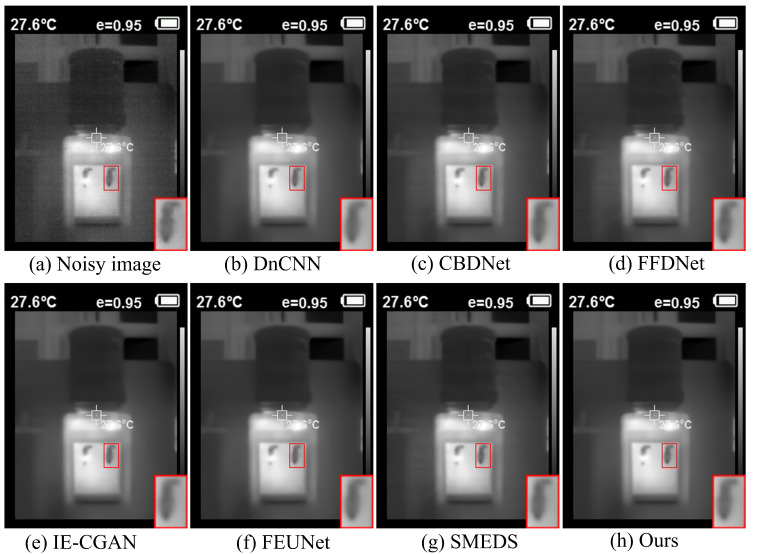
Real indoor infrared image (water dispenser).

**Figure 12:**
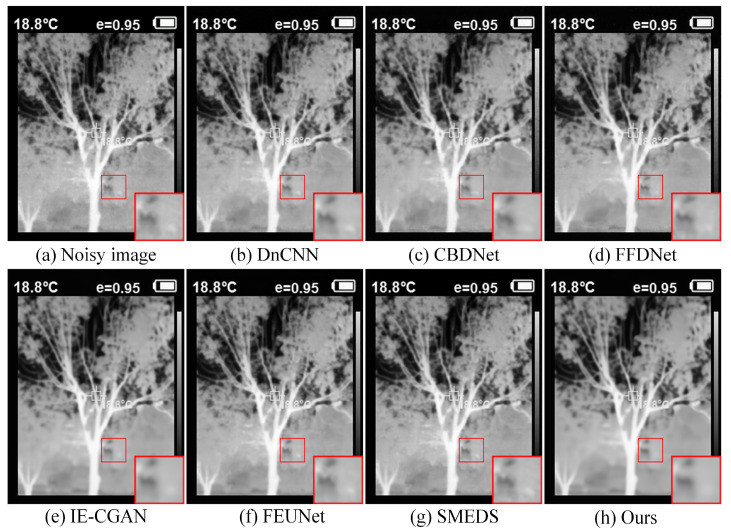
Infrared image of an outdoor scene (tree trunk).

The device used to collect real infrared scene images in this paper is the HT-18 infrared thermal imager, with a resolution of 320 × 240. When dealing with real images with unknown noise levels, the model trained under random additive white Gaussian noise levels σ = 0~25 is used for blind denoising. For fairness and simplicity, traditional methods such as BM3D are excluded in this experiment. It was observed that images with significant temperature differences often have lower noise, while images captured in indoor or non-thermal target scenes tend to have more severe and complex noise. Therefore, an indoor scene (water dispenser) and an outdoor scene (tree trunk) were selected for display. As shown in the indoor scene in Figure 11, compared with the DnCNN, IE-CGAN, and SMEDS methods, the proposed method exhibits higher contrast and clearer textures in denoising. Figure 12 shows that although all methods effectively remove noise in the outdoor scene, the proposed method demonstrates a significant advantage in removing white noise when zooming in on the texture details, thus performing better in preserving the original structure and texture information of the image.

### 3.4. Efficiency Analysis

As shown in Table 2, the performance of different models was evaluated from three aspects: runtime, the size of training parameters, and floating point operations (FLOPs). In the experiment, a 1 × 256 × 256 image was used as the input for forward propagation to assess the computational demands of the models. Although DnCNN demonstrates considerable efficiency in runtime, it performs poorly in terms of computational complexity and parameter size. Due to its numerous convolutional layers and multiple up-sampling and down-sampling operations, FEUNet has the highest computational complexity. The proposed method strikes a balance between denoising performance and resource consumption by applying structural reparameterization, making the model more lightweight.

### 3.5. Ablation Experiments

To verify the effectiveness of the proposed transfer strategy and the improvements in denoising performance brought by the edge operator and progressive multi-scale attention block (PMAB), a series of detailed experiments were designed. First, three groups of control experiments were designed on the complete model to verify the effectiveness of the transfer strategy. Control Group A used direct training on the target domain, Control Group B directly loaded pre-trained weights and trained the entire network, and Control Group C adopted a staged transfer strategy. Then, the effectiveness of different components in improving model performance was analyzed. The experiments were conducted on the infrared test set with noise level (σ = 15).

**Figure 13:**
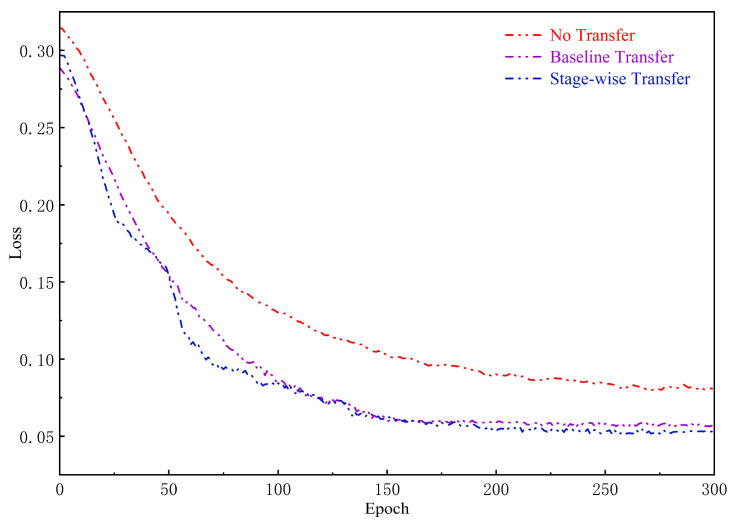
Comparison graph of the loss function.

The change in the loss function is shown in Figure 13. The comparison results show that the staged transfer strategy (Stage-wise Transfer) exhibits a significant advantage in the early stages of training, with the loss value rapidly and steadily decreasing. Throughout the training period, the loss value of this strategy is consistently the lowest. In contrast, the direct transfer strategy (Baseline Transfer) shows a lower initial loss, but as training continues, it gradually exhibits overfitting tendencies. The no-transfer strategy (No Transfer) has the highest loss value throughout the training process, indicating that this strategy has weak feature extraction capabilities on small sample datasets and poor training results. Table 3 shows that the staged transfer strategy demonstrates the best performance.

Table 4 summarizes the contributions of different components to the model’s denoising performance. From the table, it can be observed that the proposed method consistently outperforms other experimental groups across various metrics, highlighting the effectiveness of the collaboration between the Sobel edge operator and the PMAB.

### 3.6. Structural Reparameterization Experiments

To compare the performance of the model before and after structural reparameterization, four aspects were considered: runtime, the size of training parameters, floating point operations (FLOPs), and cumulative pixel deviation of denoised results. To accurately evaluate the efficiency of the method, a 1 × 256 × 256 image was used as the input for forward propagation, similar to Section 3.4. The experimental results are shown in Table 5. The results indicate that the denoising results of the network before and after structural reparameterization show almost no difference, demonstrating that structural reparameterization effectively reduces the model’s runtime and parameter size with minimal error. At the same time, the reduction in FLOPs indicates a significant improvement in computational efficiency. This lightweight design is particularly important for deploying models in resource-constrained environments, as it can significantly reduce computation and storage costs while ensuring performance.

## 4. Conclusions

This paper proposes a lightweight infrared image denoising method based on the generative adversarial network (GAN). The method integrates transfer learning with a phased feature transfer strategy to effectively address the challenges of infrared image denoising in data-limited conditions. The main conclusions are as follows:

(1) Feature Transfer and Adversarial Learning Mechanism: The generator is initially trained using a large-scale grayscale visible light image dataset, and then fine-tuned on an infrared image dataset using feature transfer techniques. The phased transfer strategy significantly improves the model’s feature representation capability in small sample environments. Through adversarial learning, the discriminator supplies the generator with precise adversarial loss, continuously optimizing the generator’s performance during the adversarial process and thereby further enhancing the generator’s feature representation capability. The experimental results show that, compared with existing methods, the proposed method demonstrates superior feature representation capability and denoising performance.
(2) Lightweight Model Design: The generator utilizes structural reparameterization technology to transform the multi-branch structure into an equivalent single-branch structure during the inference stage, which effectively reduces computational resource consumption. This design achieves an efficient lightweight model. The experimental results show that it significantly reduces computation and storage costs while maintaining denoising performance.
(3) Enhanced Edge and Texture Feature Extraction: The design of structurally reparameterized edge convolution modules and progressive multi-scale attention modules enhances the generator’s ability to extract edge and texture features, significantly improving the denoising effect.

The proposed lightweight infrared image denoising method, validated on public datasets and real-world datasets obtained from experimental equipment, effectively addresses the challenges of infrared image denoising under data-limited conditions by combining the concepts of GAN and transfer learning. It strikes a good balance between denoising performance and resource consumption, showing strong practical application value.

Although the proposed method has achieved good results in dealing with additive Gaussian white noise, it still has certain limitations when handling other types of noise, such as non-uniform stripe noise and salt-and-pepper noise. Infrared imaging is often affected by various types of noise, making mixed noise modeling an important research direction in the field of infrared image denoising. In addition, future work will focus on enhancing the model’s robustness in complex scenarios to adapt to a wider range of applications.

## Figures and Tables

**Table 1:** Average PSNR (dB)/SSIM results of different methods on AWGN images.

Model	15		25		50	
	PSNR	SSIM	PSNR	SSIM	PSNR	SSIM
BM3D [5]	32.352	0.765	30.229	0.747	25.326	0.497
DnCNN [8]	33.393	0.811	30.231	0.719	29.525	0.626
CBDNet [25]	34.281	0.826	32.928	**0.789**	30.718	0.731
FFDNet [9]	34.241	0.816	32.872	0.784	30.614	0.721
IE-CGAN [26]	34.182	0.807	32.746	0.776	30.036	0.713
FEUNet [27]	34.284	0.812	32.892	0.783	30.732	0.727
SMEDS [11]	34.332	0.817	32.938	0.786	31.038	0.731
Ours	**34.389**	**0.829**	**32.962**	0.788	**31.168**	**0.736**

**Table 2:** Comparison of computational efficiency of different denoising methods.

Model	Metric		
	Runtime (S)	Params (M)	Flops (G)
DnCNN [7]	0.316	2.546	43.561
CBDNet [25]	0.532	16.638	40.058
FFDNet [9]	0.375	1.856	8.821
IE-CGAN [26]	0.556	9.627	32.815
FEUNet [27]	0.648	6.587	82.554
SMEDS [11]	0.337	0.983	37.724
Ours	**0.226**	**0.758**	**8.715**

**Table 3:** Evaluation metrics of different transfer strategies.

Transfer Strategy	Metric	
	PSNR	SSIM
No Transfer	28.875	0.769
Baseline Transfer	32.648	0.798
Stage-wise Transfer	34.389	0.829

**Table 4:** The impact of different components on model denoising performance.

Method	Metric	
	PSNR	SSIM
No Sobel	33.875	0.806
No PMAB	32.395	0.787
No PMAB and Sobel	26.875	0.736
Ours	34.389	0.829

**Table 5:** Comparison of the results before and after structural reparameterization.

Structural Reparameterization	Metric			
	Runtime (S)	Params (M)	Flops (G)	Cumulative Pixel Deviation
No	0.289	0.826	9.856	/
Yes	0.226	0.758	8.715	1.58 × 10^−4^

## Data Availability

The data supporting the results of this study can be obtained from the corresponding author upon request.
